# Self-care practices for puerperal sepsis prevention and their determinants among postpartum women: a community-based cross-sectional study in Halaba Town, Central Ethiopia

**DOI:** 10.1017/S1463423626101571

**Published:** 2026-07-31

**Authors:** Aberash Beyene Derribow, Mebratu Demissie Senbeta, Keyredin Nuriye Metebo, Melese Gabure Shukulo, Aynalem Belay, Berhanu Semra Mulat, Mangistu Abera

**Affiliations:** 1 Midwifery, Wolkite University, Wolkite, Ethiopia; 2 Midwifery, Wachemo University, Ethiopia; 3 Public Health, Wolkite University, Ethiopia

**Keywords:** determinants, Ethiopia, puerperal sepsis, self-care practice

## Abstract

**Aim:**

This study aimed to assess self-care practices for puerperal sepsis prevention and their determinants among postpartum women in Halaba Town, Central Ethiopia.

**Background:**

Self-care is the ability to maintain health, prevent disease, and manage illness with or without the support of healthcare personnel. Current evidence indicates a high burden of puerperal sepsis in East Africa, including Ethiopia. However, evidence on self-care practices for the prevention of puerperal sepsis and their determinants in the study setting remains limited.

**Methods:**

A community-based cross-sectional study was conducted among postpartum women in Halaba Town from February 1 to March 30, 2025. Data were collected through face-to-face, interviewer-administered questionnaires. Bivariable and multivariable logistic regression analyses were performed to identify determinants of self-care practices; statistical significance was set at a p-value of less than 0.05.

**Findings:**

In the present study, 413 study participants were interviewed, resulting in a response rate of 98.3%. The prevalence of good self-care practice was 43.8% (95% CI: 39.1%–48.5%). Women’s occupation (adjusted odds ratio (AOR) = 2.01; 95% CI: 1.23–3.57), educational attainment (AOR = 2.30; 95% CI: 1.46–7.93), antenatal care (ANC) contact (AOR = 2.73; 95% CI: 1.65–4.71), and women’s knowledge (AOR = 3.30; 95% CI: 2.32–10.42) were determinants of self-care practices for puerperal sepsis prevention.

**Conclusion:**

The findings revealed that more than half of postpartum women had poor self-care practices for the prevention of puerperal sepsis. Hence, strengthening ANC contact, targeting women with low educational status and unemployed women, and improving women’s knowledge may help to enhance awareness and prevention of puerperal sepsis.

## Introduction

Self-care is the ability of individuals, families, and communities to promote health, prevent disease, maintain well-being, and cope with illness and disability, with or without the support of healthcare personnel (World Health Organisation, 2019). Postpartum sepsis is a life-threatening condition characterized by organ dysfunction caused by infection occurring during pregnancy, childbirth, or the postpartum period, as defined by the World Health Organization (WHO) (Pundir and Coomarasamy, 2016; World Health Organisation, 2017). Puerperal sepsis presents with foul-smelling vaginal discharge, abdominal tenderness, fever, and delayed uterine involution and is associated with factors such as prolonged rupture of membranes, pelvic infection, multiple vaginal examinations, and mode of delivery (World Health Organisation, 2017; Kumar and Yadav, 2020; Seboka *et al.*, 2024).

Globally, puerperal sepsis is among the preventable leading causes of maternal morbidity and mortality in both developing and developed countries (Atlaw *et al*., 2019; Plante *et al*., 2019; Kumar and Yadav, 2020). The evidence indicates that puerperal sepsis further causes pelvic infection and infertility if not treated early (World Health Organisation, 2017; Christiana *et al*., 2023). Moreover, when mothers suffer from puerperal sepsis, they are unable to care for their children, which increases the chance of early neonatal loss (Bellizzi *et al*., 2017; Fenny *et al*., 2021).

Worldwide, approximately 260,000 maternal deaths occur annually, with the majority occurring in low-and middle-income countries, particularly in Africa. Maternal infections, including puerperal sepsis, account for about 10–15% of these deaths (World Health Organisation, UNICEF, UNFPA, 2023). In developing countries, puerperal sepsis continues to be a major contributor to maternal morbidity and mortality, responsible for about 15% of maternal deaths (Berhan and Berhan, 2014; Lindtjørn *et al*., 2017; Souza *et al*., 2024).

In African countries, the prevalence of puerperal sepsis ranges from 2% to 24% (Bishaw *et al*., 2023; Bolanle *et al*., 2024; Halil *et al*., 2025), often leading to surgical complications, multi-organ damage, and death (Kumar and Yadav, 2020). Evidence shows that sepsis requires urgent medical attention, early treatment, and resuscitation. Healthcare personnel should promptly diagnose and treat sepsis within an hour to prevent organ damage and improve survival, even in the absence of fever (Plante *et al*., 2019).

Previous findings indicate that, beyond healthcare provider interventions, mothers’ self-care plays a significant role in promoting well-being during the postpartum period. Self-care refers to a mother’s ability to care for herself through good hygiene, nutrition, exercise, rest, and attention to physical appearance (World Health Organisation, 2019; Barkin *et al*., 2020). However, evidence revealed that mothers generally have fewer self-care practices for the prevention of puerperal sepsis. Approximately 54.4% to 75% of postpartum women demonstrated poor self-care practices (Sultana *et al*., 2018; Hassan *et al*., 2021; Abdel-fattah *et al*., 2022; Nchimbi and Joho, 2022; Teferi *et al*., 2024).

A study in Egypt showed that more than half of the participants reported poor self-care practices (Abdel-fattah *et al*., 2022). Similarly, in Bangladesh, puerperal sepsis self-care practices were poor (Sultana *et al.*, 2018). In Tanzania, more than four-fifths of mothers had poor self-care practices (Nchimbi and Joho, 2022). Likewise, a study in Ethiopia indicated more than half of postpartum mothers had poor puerperal sepsis prevention practices (Teferi *et al*., 2024). Self-care practices for puerperal sepsis prevention in the postpartum period include a range of behaviors aimed at reducing infection risk and promoting recovery. These include maintaining perineal hygiene and proper handwashing, regular changing of sanitary pads, wound care and monitoring of vaginal discharge, adequate nutrition and fluid intake, postpartum physical activity, adherence to postpartum care visits, and recognition of danger signs such as fever and abnormal vaginal discharge (World Health Organisation, 2019; Nchimbi and Joho, 2022; Teferi *et al*., 2024; Abera *et al*., 2025a).

In Ethiopia, the maternal mortality ratio decreased from 401 in 2017 to 267 in 2020; however, further improvements are needed to achieve the target of 70 per 100,000 live births by 2030, as approximately 12,000 mothers die each year. Moreover, thirteen percent of these deaths are attributed to puerperal sepsis (Berhan and Berhan, 2014; Ministry of Health, 2017; Lindtjørn *et al*., 2017; Souza *et al*., 2024). By 2026, Ethiopia aims to reduce maternal mortality to 199 per 100,000 live births to achieve Sustainable Development Goal 3.1 by 2030 through the reduction of direct causes, including puerperal sepsis (Ministry of Health, 2022). Hence, to achieve this plan, addressing contributors to maternal morbidity and mortality is essential.

In Ethiopia, primary healthcare is delivered through the Health Extension Program, where Health Extension Workers provide community-based maternal health services, including health education, home visits, and antenatal and postpartum follow-up. These services are important for promoting hygienic postpartum practices and preventing maternal complications such as puerperal sepsis.

Even though a high burden of puerperal sepsis exists in most developing settings, existing literature suggests that self-care practices for the prevention of puerperal sepsis in Central Ethiopia remain underexplored, with limited empirical evidence documenting their nature and influence on maternal health outcomes. This study aimed to assess self-care practices for puerperal sepsis prevention and their determinants among postpartum women in Halaba Town, Central Ethiopia.

## Methods and materials

### Study setting and period

**Figure 1.**
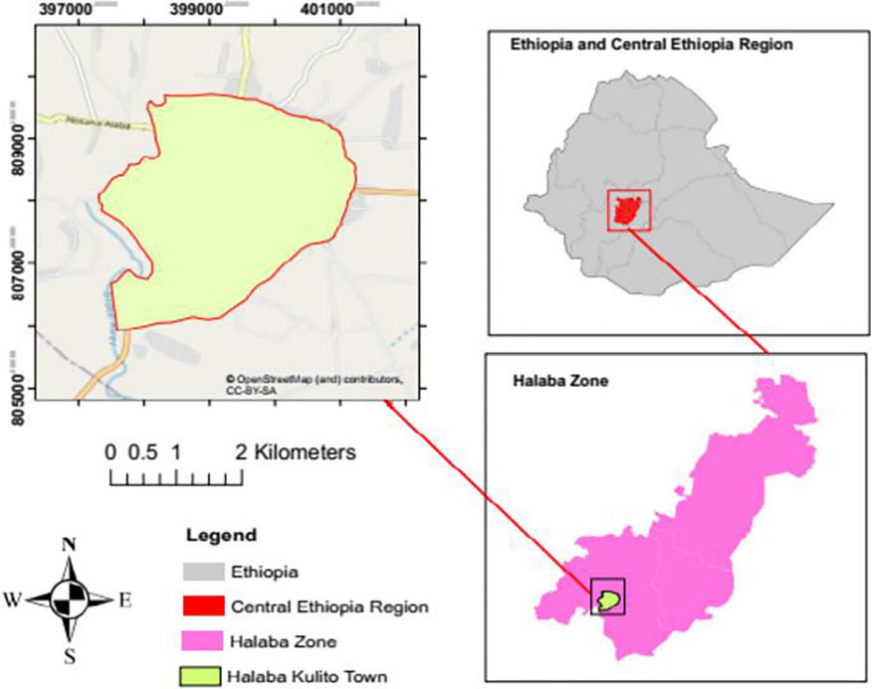
Map showing the location of Halaba Town in Halaba Zone, Central Ethiopia, 2025.

The Halaba Zone is located in the Central Ethiopia region. Based on the 2017 census conducted by the Central Statistical Agency of Ethiopia, Halaba Zone has a total population of 232,325, of whom 117,291 are men, and 115,034 are women. Halaba Kulito Town is located in Central Ethiopia and serves as the administrative centre of Halaba Zone. It is approximately 220 km south of Addis Ababa and is situated at 7°18′44″ N, 38°05′21″ E. The town consists of five kebeles and has one general hospital, one health centre, and five health posts (Figure 1). A community-based cross-sectional study was conducted in Halaba Town from February 1 to March 30, 2025.

### Study population and their eligiblity criteria

**Source population:**

All postpartum women residing in Halaba Town.

**Study population:**

All postpartum women residing in Halaba Town who had given birth within the first six weeks during the study period.

**Inclusion criteria:**

All postpartum women within the first six weeks after delivery who had lived in the town for at least six months during the study period and who were willing to participate in the study were included.

**Exclusion criteria:**

Postpartum women who were critically ill during data collection were excluded from the study.

### Sample size determination

The sample size was calculated using a single population proportion formula by considering 45.6% (prevalence of self-care practices for puerperal sepsis prevention) (Teferi *et al*., 2024), with a 95% confidence interval and a 0.05 margin of error. $\begin{array}{ll} n & =\frac{\left(Z\alpha /2)2p(1-p\right)}{d2}=n=\frac{\left(1.96\right)2*(0.456)*(0.544)}{(0.05)2}\\ =381.19\approx \;381 \end{array}$

The sample size was rounded up to 382 to ensure adequacy and correct arithmetic calculation of the sample size. After adding a 10% non-response rate, the final sample size was **420.**

In Halaba town, there are five kebeles, and all were enrolled in this study. In the town, the estimated number of deliveries within the first 6 weeks in each Kebele was 793, from the logbooks of health extension workers. The sample was allocated proportionally to each kebele (Denbefam 85, Murasa ber 68, Lenda ber 119, Mehal Arad 93, and Wanja ber 55). A systematic random sampling technique was employed to select study participants. The sampling interval (K) was calculated using *K* = *N*/*n*, which yielded approximately two. A simple random sampling method was used to select the first participant (starting point) at the household level from the first two eligible postpartum women in each kebele. Thereafter, every second postpartum woman was selected until the required sample size was reached. If more than one eligible postpartum woman was found in a selected household, one was selected randomly.

### Study variables

The dependent variable was self-care practices for the prevention of puerperal sepsis. The independent variables included sociodemographic factors (age, ethnicity, residence, religion, marital status, educational level, occupation, and family wealth index), obstetric related factors (parity, number of antenatal care (ANC) contacts, place of ANC contact, gestational age at first ANC visit, and place of birth), and postpartum behavioural and health-related factors (postpartum danger signs, nutrition, exclusive breastfeeding, immunization schedule, hygiene practices, physical exercise, family planning use, knowledge of puerperal sepsis, and counselling received on its symptoms).

Additionally, exclusive breastfeeding and immunization schedule were included as they are key components of routine postpartum counselling and may reflect a woman’s engagement with recommended postpartum care services. These practices may also serve as proxies for overall health-seeking behaviour, which could influence adherence to self-care practices for the prevention of puerperal sepsis.

### Operational definitions

***Self-care*** is the capability of women to promote health, prevent disease, and maintain well-being with or without the support of the healthcare personnel (World Health Organisation, 2019).

According to the WHO, puerperal sepsis is a genital tract infection occurring from membrane rupture or labour up to 42 days postpartum, marked by pelvic pain, fever, abnormal vaginal discharge, foul odour, or delayed uterine involution (World Health Organisation, 2017).

In this study, postpartum mothers scoring at or above the mean on self-care practice questions were considered to have **good self-care practices**, whereas those scoring below the mean were considered to have **poor self-care practices** for preventing puerperal sepsis (Sultana *et al*., 2018; Nchimbi and Joho, 2022; Teferi *et al*., 2024).

**Family income** was scored based on assets and housing characteristics, analysed using Principal Component Analysis (PCA), and divided into three categories: poor (1st–2nd quintiles), medium (3rd quintile), and rich (4th–5th quintiles) (Chakraborty *et al.*, 2016; Abera *et al*., 2025b; Tengo *et al*., 2025a).

### Data collection tool and procedures

The tool was adapted from previous literature for collecting data (Sultana *et al.*, 2018; Nchimbi and Joho, 2022; Teferi *et al*., 2024). The questionnaire was prepared in English, translated into the local language, and back-translated to English to ensure consistency. The tools contained socio-demographic, obstetrics-related factors, women’s knowledge of puerperal sepsis, and self-care practices to prevent puerperal sepsis.

Self-care practices were assessed using sixteen practice-related questions, with the overall mean computed and dichotomized: mothers scoring the mean and above were coded ‘1’ for good self-care practices, and those scoring below the mean were coded ‘0’ for poor self-care practices. This research used a validated tool, pre-tested for clarity and reliability, with content validity checked by field experts. The self-care practice scale consisted of sixteen items and demonstrated excellent internal consistency, with a Cronbach’s alpha of 0.97. The high alpha value reflects strong reliability of the scale rather than item redundancy, as the items assessed different aspects of postpartum self-care practices for the prevention of puerperal sepsis. Data were collected through face-to-face household interviews by five BSc nurses and one MSc midwife supervisor who spoke the local languages.

### Data quality management

The questionnaire was pre-tested on 5% of the sample size at Sinbita kebele prior to the actual data collection to ensure clarity, consistency, and appropriateness of the data collection tool. Following the pretest, necessary modifications were made, and the pretest data were excluded from the final analysis. Two days of training were provided for data collectors and supervisors on the objectives of the study, interviewing techniques, ethical considerations, and proper completion of the questionnaire to ensure data quality and consistency throughout the data collection process.

The supervisors closely monitored data collection, checked questionnaires daily for completeness and consistency, and provided immediate feedback to data collectors on time. After data collection, all questionnaires were rechecked before data entry. Double data entry was then performed using the EpiData software system. In addition, data cleaning was performed using simple frequencies and graphical methods, including box plots, to identify missing values and outliers.

### Statistical analysis

After the data were coded and entered into EpiData version 4.60, they were exported to SPSS version 27 for analysis. The household wealth index was computed using principal component analysis (PCA) based on 15 household asset items. The adequacy of the data for PCA was assessed using the Kaiser–Meyer–Olkin (KMO) measure and Bartlett’s test of sphericity. Components with eigenvalues greater than 1 were retained, and the first principal component was used to generate the wealth index, which was categorized into quintiles. These quintiles were further recategorized into three categories: poor (1st–2nd quintiles), medium (3rd quintile), and rich (4th–5th quintiles). Descriptive statistics, including frequencies, percentages, means, and standard deviations, were computed to summarize the data. Binary logistic regression was first performed to identify candidate variables, and variables with *p* ≤ 0.25 were entered into the multivariable logistic regression model.

The adequacy of the multivariable model was assessed using events per variable, with a minimum threshold of ≥10 considered acceptable. Model fitness was confirmed by the Hosmer and Lemeshow test (*p* = 0.527), omnibus tests (*p* < 0.001), and multicollinearity checked via variance inflation factor (maximum = 2.91) and tolerance (minimum = 36.9%) for predictors of self-care practice for puerperal sepsis prevention, indicating no multicollinearity issues. Additional model diagnostics, including assessment of influential observations using Cook’s distance and leverage values, confirmed that no single observation unduly influenced the model estimates. Statistical significance was declared at a p-value less than 0.05, and adjusted odds ratios (AORs) with 95% confidence intervals were used to report associations. Finally, the findings were reported in narration and tables.

## Results

### Socio-demographic characteristics of the respondents

A total of 413 participants were interviewed, resulting in a response rate of 98.3%. Of the respondents, 183 (44.3%) were aged 26–35 years (30.1 ± 7.5 SD). Approximately 120 participants (29.0%) had attained primary education, and 159 (38.6%) reported a high family income (Table 1).

**Table 1.** Socio-demographic characteristics of postpartum women in Halaba Town, Central Ethiopia, 2025 (*n* = 413) Table 1 long description.

Study variables	Category	Frequency	Percent (%)
Age of the respondents in years	15–25	117	28.3
	26–35	183	44.3
	>35	113	27.4
Marital status	Married	404	97.8
	Unmarried	9	2.2
Educational attainment	No formal education	92	22.3
	Primary school	120	29.0
	Secondary school	89	21.5
	Diploma and above	112	27.2
Women’s occupation	Unemployed	123	29.7
	Private work	131	31.7
	Government employee	159	38.6
Family income	Low	119	28.8
	Medium	135	32.6
	High	159	38.6

*Note:* Unemployed means women who were not engaged in formal government or private employment, including housewives and those engaged in informal or unpaid activities.

### Obstetrics-related factors of the study respondents

More than half of the study participants (59.8%) were multiparous. A total of 159 (38.4%) respondents had ≥8 ANC contacts, with a mean ANC contact of 5.4 ± 3.0 SD, and 76.8% of respondents started their follow-up in early pregnancy (first trimester).

All respondents reported receiving at least one form of postpartum counselling from health care providers after delivery. However, counselling was delivered on different components rather than as a uniform package, and coverage varied across specific topics. The most commonly reported counselling components were child immunization (98.5%) and family planning (98.3%), followed by breastfeeding (97.8%) and postpartum danger signs (96.4%). In contrast, counselling on postpartum exercise was very low (5.8%).

Regarding specific symptoms of puerperal sepsis, less than half of the women reported receiving counselling on fever (47.6%), foul-smelling vaginal discharge (43.1%), and abdominal tenderness (16.3%). Furthermore, 245 (59.4%) of postpartum women were knowledgeable regarding puerperal sepsis (Table 2).

**Table 2.** Obstetrics-related factors of postpartum women in Halaba Town, Central Ethiopia, 2025 (*n* = 413) Table 2 long description.

Study variables	Category	Frequency	Percent (%)
Parity	Primiparous	166	40.2
	Multiparous	247	59.8
Number of ANC contacts	<8 ANC contact	254	61.6
	≥8 ANC contact	159	38.4
Place of ANC contact	Hospital	211	51.0
	Health centre	171	41.4
	Private clinic	31	7.6
Trimester of first ANC contact	First trimester	317	76.8
	Second trimester	96	23.2
The current baby’s place of birth	Hospital	323	78.2
	Health centre	86	20.8
	Home	4	1
**Counselling received from health care providers**			
Postpartum danger sign	Yes	398	96.4
	No	15	3.6
About nutrition	Yes	317	76.8
	No	96	23.2
Regarding breastfeeding	Yes	404	97.8
	No	9	2.2
Child immunization	Yes	407	98.5
	No	6	1.5
Personal hygiene	Yes	300	73
	No	113	27
Postpartum exercise	Yes	24	5.8
	No	389	94.2
Family planning services	Yes	406	98.3
	No	7	1.7
Women’s knowledge of puerperal sepsis	Knowledgeable	245	59.4
	Not Knowledgeable	168	40.6
**Counselling on symptoms of puerperal sepsis**			
Fever	Yes	197	47.6
	No	216	52.4
Foul-smelling vaginal discharge	Yes	178	43.1
	No	235	56.9
Abdominal tenderness	Yes	67	16.3
	No	346	83.7

**ANC:** Antenatal care.

### Self-care practices to prevent puerperal sepsis

The overall prevalence of good self-care practices for the prevention of puerperal sepsis was 43.8% (95% CI: 39.1%–48.5%). In addition to the overall self-care classification, individual self-care practices were assessed. The most commonly reported practices included perineal hygiene and handwashing practices, health-seeking and follow-up practices, and wound care and vaginal discharge monitoring practices, which were practiced by most respondents. In contrast, practices such as sanitary pad management, postpartum physical activity, nutritional and fluid intake, and recognition and response to danger signs were less frequently reported.

### Determinants of self-care practices to prevent puerperal sepsis

In binary logistic regression analysis, candidate variables were selected for multivariable logistic regression at a *p*-value of ≤0.25. Variables such as educational attainment, occupation, family income, parity, ANC contacts, and women’s knowledge regarding puerperal sepsis were identified as candidate variables for multivariable logistic analysis.

These candidate variables were entered into a multivariable logistic regression using the backward method to determine the final determinants of women’s self-care practices to prevent puerperal sepsis while controlling for potential confounders.

In multivariable analysis, four variables, such as educational attainment, occupation, ≥8 ANC contacts, and women’s knowledge, were identified as determinants of self-care practices to prevent puerperal sepsis after adjusting for confounders in the final model.

Postpartum women who were government employees were two times more likely (AOR = 2.01; 95% CI: 1.23–3.57) to have good self-care practices to prevent puerperal sepsis than those who were unemployed.

Postpartum women who had completed a diploma or higher education were 2.30 times more likely (AOR = 2.30; 95% CI: 1.46–7.93) to have good self-care practices to prevent puerperal sepsis than those who did not attend formal education.

Postpartum women who attended ≥8 ANC contacts were 2.73 times more likely (AOR = 2.73; 95% CI: 1.65–4.71) to have good self-care practices compared to their counterparts.

Postpartum mothers who were knowledgeable were 3.30 times more likely to have good self-care practices for puerperal sepsis prevention compared to those who were not knowledgeable about puerperal sepsis (AOR = 3.30; 95%CI:2.32–10.42), indicating a statistically significant association. However, the relatively wide confidence interval suggests limited precision, likely due to sparse data in some cells; therefore, the result should be interpreted with caution. Variables such as private work, primary education, completion of secondary education, parity, and family income were not statistically significant after adjustment in the final model (Table 3).

**Table 3.** Bivariable and multivariable analysis of self-care practices to prevent puerperal sepsis in Halaba Town, Central Ethiopia, 2025 (*n* = 413) Table 3 long description.

Self-care practices to prevent puerperal sepsis						
**Variables**	**Good (*n*)**	**Poor (*n*)**	**COR (95% CI)**	***P*-value**	**AOR (95% CI)**	***P*-value**
**Occupation**						
Unemployed	43	80	**1**		**1**	
Private work	58	73	1.47 (0.89–3.54)	0.120	1.20 (0.98–3.58)	0.213
Government employee	80	79	1.90 (1.10–3.20)	0.008*	2.01(1.23–3.57)	0.004**
**Educational attainment**						
No formal education	29	63	**1**		**1**	
Primary education	46	74	1.35 (1.13–3.57)	0.030*	1.20 (0.30–3.46)	0.92
Secondary completed	37	52	1.55 (1.10–3.74)	0.028*	1.30 (0.73–3.15)	0.240
Diploma and above	69	43	3.48 (1.71–8.21)	0.001*	2.30 (1.46–7.93)	0.007**
**ANC contact**						
<8 Contact	78	176	**1**		**1**	
≥8 contacts	103	56	4.15 (2.33–9.21)	<0.001*	2.73 (1.65–4.71)	0.001**
**Parity**						
Primiparous	56	110	**1**		**1**	
Multiparous	125	122	2.01 (1.17–3.76)	0.015*	1.8 (0.91–4.59)	0.082
**Family income**						
Low	47	72	**1**		**1**	
Medium	42	93	0.69 (0.52–1.57)	0.410	0.40 (0.30–1.19)	0.370
High	92	67	2.10 (1.05–4.95)	0.032*	1.32 (0.76–3.62)	0.120
**Women’s knowledge**						
Not knowledgeable	37	131	**1**		**1**	
Knowledgeable	144	101	5.05 (2.37–11.9)	<0.001*	3.30 (2.32–10.42)	0.001**

*Note:* **ANC:** Antenatal Care, **AOR:** Adjusted Odds Ratio, **COR:** Crude Odds Ratio, **CI:** Confidence Interval, **1:** Reference,*: significant candidate variables for multivariable analysis at *p* ≤ 0.25,**: statistically significant at *p* < 0.05.

## Discussion

This community-based cross-sectional study assessed self-care practices for the prevention of puerperal sepsis and identified their determinants. Poor self-care practices during the postpartum period remain a major contributor to maternal morbidity and mortality, increasing the risk of postpartum complications among mothers. The overall prevalence of good self-care practices for the prevention of puerperal sepsis was 43.8% (95% CI: 39.1%–48.5%). This indicates that more than half of the respondents had poor self-care practices for the prevention of puerperal sepsis. This finding is consistent with a study conducted in Arba Minch, Town (45.6%) (Teferi *et al*., 2024). The result is higher compared to studies conducted in Egypt (25.3%) and Tanzania (11.4%) (Hassan *et al*., 2021; Nchimbi and Joho, 2022). The differences in prevalence between the current study and those conducted in Egypt and Tanzania may be attributed to methodological and contextual variations. The present study was community-based, whereas the studies from Egypt and Tanzania were facility-based, which may introduce selection bias and differences in exposure to maternal health services and counselling. In addition, although the included studies assessed self-care practices for puerperal sepsis prevention using broadly comparable operational definitions, minor differences in scoring methods and cut-off points may have influenced prevalence estimates. Variations in study period may also reflect changes in maternal health service coverage and awareness over time. Other contributing factors may include differences in counselling on postpartum danger signs, women’s knowledge of puerperal sepsis, and variations in educational status and awareness of preventive practices.

In this study, government employees, diploma and above educational attainment, ≥8 antenatal contacts, and women’s knowledge regarding puerperal sepsis were determinants of self-care practices to prevent puerperal sepsis. Postpartum women who were government employees were 2.01 times more likely to have good self-care practices to prevent puerperal sepsis. Beyond the relative association, absolute differences further highlighted disparities in self-care practices across occupational groups, with government-employed women reporting a higher proportion of good self-care practices (44.2%) compared with unemployed women (23.8%) and those engaged in private work (32%). This finding suggests that occupation-related differences in access to information and health services may influence postpartum self-care behaviors. The possible justification is that women who are government employees have information regarding puerperal sepsis prevention as well as access to reproductive health care services compared to unemployed women (Akhter *et al*., 2025). This finding is similar to a study conducted in Tanzania (Nchimbi and Joho, 2022). Women with a diploma or higher were 2.30 times more likely to have good self-care practices to prevent puerperal sepsis than their counterparts. These findings are consistent with studies conducted in Bangladesh, Tanzania, Arba Minch Town, and Egypt (Sultana *et al.*, 2018; Hassan *et al*., 2021; Nchimbi and Joho, 2022; Teferi *et al*., 2024). The evidence revealed that mothers’ knowledge increased as their education status increased (Sultana *et al*., 2018; Nchimbi and Joho, 2022).

The mothers who attended ≥8 ANC contacts were 2.73 times more likely to have good self-care practices to prevent puerperal sepsis compared to their counterparts. During antenatal follow-up, the chance to receive information on puerperal sepsis and its prevention from healthcare providers increases, which enhances their self-care practices. The current findings revealed that, as antenatal contacts increase, the self-care practice to prevent puerperal sepsis also increases (Nchimbi and Joho, 2022; Lee *et al*., 2024). Furthermore, mothers who attend antenatal care during pregnancy could be more aware of postpartum danger signs and self-care during the postpartum period (Abera *et al*., 2025a; Tengo *et al*., 2025b).

Postpartum women who were knowledgeable were 3.3 times more likely to have good self-care practices to prevent puerperal sepsis as compared to those who were not knowledgeable. This finding aligns with studies conducted in Tanzania, Arba Minch, and Egypt (Hassan *et al*., 2021; Nchimbi and Joho, 2022; Teferi *et al*., 2024). The possible explanation may be that knowledgeable women are more likely to engage in health-protective behaviors and adopt recommended self-care practices during the puerperal period (Bishaw *et al*., 2022; Abera *et al*., 2025a). Further, higher levels of knowledge may be associated with improved self-care practices among mothers (Mostafa *et al*., 2020).

Furthermore, this study highlighted important gaps in the content of postpartum counselling provided to mothers. Although counselling on general personal hygiene was relatively common (73%), essential components related to puerperal sepsis prevention, such as counselling on abdominal tenderness as a danger sign (16.3%), were considerably less emphasized. In addition, counselling on postpartum exercise was notably low (5.8%), suggesting that important aspects of physical recovery received limited attention. These findings indicate that postpartum counselling may lack comprehensiveness and appear to place greater emphasis on general hygiene practices than on critical danger signs and recovery-related information. Such variations in counselling content may have important implications for maternal knowledge and self-care practices, as inconsistent counselling may limit women’s ability to effectively engage in optimal postpartum self-care within the self-care framework (Yeneabat *et al*., 2022; Abera *et al*., 2025a). Hence, these results indicated gaps in self-care practices to prevent puerperal sepsis among mothers in Halaba Town, Central Ethiopia. The findings suggest that enhancing health education during postpartum visits is crucial. Improving ANC quality, women’s education, and counselling on postpartum danger signs can enhance self-care practices and reduce maternal complications, morbidity, and mortality.

### Strengths and limitations of the study

The study employed a community-based cross-sectional design with an adequate sample size and a response rate of 98.3%, enhancing the reliability and representativeness of the findings. Potential biases were minimized through clear objectives, pretested questionnaires, training of data collectors and supervisors, random sampling, and statistical adjustment for confounding variables. Ethical guidelines were followed to ensure unbiased participation.

However, this study has several limitations that should be considered when interpreting the findings. First, the classification of self-care practices was based on mean-based dichotomization of the outcome, which may limit comparability with other studies and influence the estimated prevalence of good and poor practices. Second, recall bias may have affected participants’ responses regarding postpartum self-care practices, potentially leading to either overestimation or underestimation of actual behaviors. Third, social desirability bias may have been introduced due to the use of face-to-face interviews, which could have resulted in over-reporting of recommended practices. In addition, the cross-sectional design of the study limits the ability to establish causal relationships, and the observed associations, such as those involving knowledge, antenatal care, and counselling, may be subject to confounding or reverse causality. The exclusion of critically ill postpartum women may have led to an underestimation of poor self-care practices. Furthermore, because the study was conducted in Halaba Town, the findings may not be generalizable to rural populations or other regions of Ethiopia. Finally, the use of health extension worker logbooks as the sampling frame may have introduced selection bias if some eligible women were not fully registered.

### Conclusion

The present findings indicated that more than half of the respondents had poor self-care practices for preventing puerperal sepsis. Maternal educational attainment, occupational status, ANC contacts, and knowledge of puerperal sepsis were associated with self-care practices. Modifiable factors may therefore be associated with improved self-care practices among postpartum mothers. Hence, strengthening ANC follow-up, focusing on women with low educational attainment and unemployed women, and improving women’s knowledge may help enhance awareness and prevention of puerperal sepsis.

### Recommendations

The following recommendations were given based on the findings: It is encouraged to provide targeted health education on puerperal sepsis and its prevention during ANC contact, using simple and culturally appropriate materials to improve women’s self-care practices. Further, at the community level, community-based campaigns focusing on mothers with lower educational levels should be designed, and health extension workers should be encouraged to disseminate information at the household level to enhance self-care practices for preventing puerperal sepsis.

Moreover, targeted strengthening of postpartum counselling is recommended, particularly focusing on improving the delivery of key puerperal sepsis danger signs, such as abdominal tenderness, which was reported by only a small proportion of women. In addition, health extension workers and maternal health providers should be trained to ensure standardized and comprehensive postpartum counselling that includes essential self-care components beyond general hygiene practices, such as postpartum exercise and recognition of danger signs. Strengthening these areas may improve women’s knowledge and subsequent self-care practices. In addition, further qualitative studies are recommended to explore barriers and facilitators of postpartum self-care practices in depth.

## Data Availability

Data that support the findings are available from the corresponding author upon a reasonable request (Email: aberashbey217@gmail.com).

## Table 1. Long description

A table with four columns and five rows, detailing socio-demographic characteristics of postpartum women in Halaba Town, Central Ethiopia, 2025. The columns are labeled Study variables, Category, Frequency, and Percent (%). The rows are grouped by different study variables: Age of the respondents in years, Marital status, Educational attainment, Women’s occupation, and Family income. Each row provides categories, frequencies, and percentages for each variable. For example, the Age of the respondents in years is divided into three categories: 15-25, 26-35, and over 35, with frequencies of 117, 183, and 113, and percentages of 28.3%, 44.3%, and 27.4%, respectively. The table provides a detailed breakdown of the socio-demographic characteristics of the respondents.

Navigate back to Table 1..

## Table 2. Long description

A table summarizing obstetrics-related factors of postpartum women in Halaba Town, Central Ethiopia, 2025. The table has 14 rows and 3 columns. The columns are labeled Study variables, Category, Frequency, and Percent. The rows are grouped into sections: Parity, Number of ANC contacts, Place of ANC contact, Trimester of first ANC contact, The current baby’s place of birth, Counselling received from health care providers, Women’s knowledge of puerperal sepsis, and Counselling on symptoms of puerperal sepsis. Each row lists specific categories and their corresponding frequency and percent values. Notable categories include Parity with Primiparous and Multiparous, Number of ANC contacts with less than 8 and 8 or more, Place of ANC contact with Hospital, Health center, and Private clinic, Trimester of first ANC contact with First trimester and Second trimester, The current baby’s place of birth with Hospital, Health center, and Home, Counselling received from health care providers on various topics, Women’s knowledge of puerperal sepsis with Knowledgeable and Not Knowledgeable, and Counselling on symptoms of puerperal sepsis including Fever, Foul-smelling vaginal discharge, and Abdominal tenderness.

Navigate back to Table 2..

## Table 3. Long description

A table with 10 rows and 7 columns comparing self-care practices to prevent puerperal sepsis by various variables. The columns are labeled as Variables, Good (n), Poor (n), COR (95% CI), P-value, AOR (95% CI), and P-value. The variables include Occupation, Educational attainment, ANC contact, Parity, Family income, and Women’s knowledge. Each row provides data for these variables, showing the number of good and poor practices, the crude odds ratio (COR) with 95% confidence interval (CI), the P-value for COR, the adjusted odds ratio (AOR) with 95% CI, and the P-value for AOR. Notable trends include higher odds of good self-care practices among government employees, those with a diploma and above, those with more ANC contacts, and those who are knowledgeable about puerperal sepsis.

Navigate back to Table 3..

## References

[ref1] Abdel-fattah N , Abdel-Moniem E and Farrag RA (2022) Knowledge and practice of postpartum mothers regarding. Indonesian Journal of Global Health Research 4(2), 323–330.

[ref2] Abera M , Tetema MD , Zeleke FT , Metebo KN , Derribow AB , Gelmesa MO , Benti Terefe A , Gudeta TG , Dabala O , Weldeyohans MA , Gabure Shukulo M and Senbeta MD (2025a) Knowledge of puerperal sepsis, self-care practices of puerperal sepsis prevention and associated factors among postpartum women in the Gurage zone, Central Ethiopia: Multicenter study. SAGE Open Medicine 13(2), 1–13. 10.1177/20503121251387974 PMC12559680 41164238

[ref3] Abera M , Belay A , Derribow AB , Bacha G and Belina S (2025b) Self-management practice of minor pregnancy disorders and associated factors among pregnant women attending antenatal clinics at Tulu Bolo General Hospital, Ethiopia. SAGE Open Nursing 11(1), 1–14. 10.1177/23779608251345324 PMC12123115 40453575

[ref4] Akhter S , Khatun F , Afrin F , Akter A , Halder CR , Biswas RK and Dey SK (2025) Factors influencing health promotion behavior on puerperal sepsis among postpartum mothers. BMC Pregnancy and Childbirth 25(1), 273. 10.1186/s12884-025-07275-y 40069706 PMC11900515

[ref5] Atlaw D , Woldeyohannes D and Berta M (2019) Puerperal sepsis and its associated factors among mothers in University of Gondar referral hospital, Ethiopia, 2017 (October). Available at 10.15406/ipcb.2019.05.00175

[ref6] Barkin JL , Wisner KL and Drive L (2020) The role of maternal self-care in new motherhood. HHS Public Access 29(9), 1050–1055. 29(9), pp. 1050-1055. 10.1016/j.midw.2012.10.001.The PMC7081756 23415369

[ref7] Bellizzi S , Bassat Q , Ali MM , Sobel HL , Temmerman M and Simeoni U (2017) Effect of puerperal infections on early neonatal mortality: A secondary analysis of six demographic and health surveys. PLoS One 12(1), 1–9. 10.1371/journal.pone.0170856 PMC5266333 28122046

[ref8] Berhan Y and Berhan A (2014) Causes of maternal mortality in Ethiopia: A significant decline in abortion related death. Ethiopian Journal of Health Sciences 24(8), 15–28. 10.4314/ejhs.v24i0.3S PMC4249203 25489180

[ref9] Bishaw KA , Sharew Y , Beka E , Aynalem BY , Zeleke LB , Desta M , Kassie B , Amha H , Eshete T , Tamir W , Bantigen K , Mulugeta H , Ferede AA and Bitewa YB (2023) Incidence and predictors of puerperal sepsis among postpartum comprehensive specialized hospital, northwest Ethiopia: A prospective cohort study. Frontiers in Global Womens Health 4, 1–10. p.966942. 10.3389/fgwh.2023.966942. PMC9902590 36760237

[ref10] Bishaw KA , Worku S and Tilahun M (2022) Prevention of puerperal sepsis in northwest Ethiopia: Knowledge and practice of postpartum women; A multicenter cross-sectional study. SAGE Open Medicine 10, 1–9. 10.1177/20503121221085842 PMC8972931 35371488

[ref11] Bolanle GO , Ojo AO , Olawade DB , Aluko JO and Esan DT (2024) Prevalence and outcome of puerperal sepsis among mothers in Nigeria: A five-year retrospective study. Women and Children Nursing 2(3), 68–73. 10.1016/j.wcn.2024.08.001

[ref12] Chakraborty NM , Fry K , Behl R and Longfield K (2016) Simplified asset indices to measure wealth and equity in health programs: A reliability and validity analysis using survey data from 16 countries. Global Health: Science and Practice 4(1), 141–154. 27016550 10.9745/GHSP-D-15-00384 PMC4807755

[ref13] Christiana ME , Eunice AS , Laraba K , Lishikah A , Tongdima LJ , Nanko IG , Mary RS and Nanmi Z (2023) Factors influencing and the effect of puerperal sepsis among postpartum women in Vom Christian Hospital, Plateau State, Nigeria. Asian Journal of Research in Nursing and Health 6(1), 352–360.

[ref14] Fenny AP , Otieku E , Akufo C , Obeng-Nkrumah N , Asante FA and Enemark U (2021) The financial impact of puerperal infections on patients, carers and public hospitals in two regions in Ghana. International Journal of Gynecology and Obstetrics 154(1), 49–55. 10.1002/ijgo.13509 33275780 PMC8246964

[ref15] Halil HM , Abdo RA , Hassen AA and Ebro SO (2025) The prevalence and determinants of puerperal sepsis among postpartum women at Hadiya Zone, Ethiopia. Journal of Midwifery and Reproductive Health 13(2), 4785–4792. 10.22038/jmrh.2024.72442.2123

[ref18] Kumar N and Yadav A (2020) Puerperal sepsis and maternal outcome in developing countries: An observational study. International Journal of Community Medicine and Public Health 7(12), 4978–4985.

[ref19] Lee S , Adam E , Kanyike AM , Wani S , Kasibante S , Mukunya D and Nantale R (2024) Compliance with the WHO recommended 8+ antenatal care contacts schedule among postpartum mothers in eastern Uganda: A cross-sectional study. PLoS One 19(12), 1–13. 10.1371/journal.pone.0314769 PMC11627358 39652571

[ref20] Lindtjørn B , Mitiku D , Zidda Z and Yaya Y (2017) Reducing maternal deaths in Ethiopia: Results of an intervention programme in Southwest Ethiopia. PLoS ONE 12(1), 1–18. 10.1371/journal.pone.0169304 PMC5207510 28046036

[ref17] Ministry of Health (2017) Maternal Health Overview of maternal health in Ethiopia. Available at https://www.moh.gov.et/en/initiatives-4-col/Maternal_Health?language_content_entity=en 6–11.

[ref21] Ministry of Health (2022) National Guideline on Prevention and Management of Postpartum Hemorrhage (May). Ethiopia: Addis Ababa, pp. 1–44.

[ref16] Mohammed Hassan RH , Mohamed HA and Solimen HA (2021) Knowledge and practices of postpartum mothers regarding prevention of puerperal sepsis. Minia Scientific Nursing Journal 9(1), 933–939.

[ref22] Mostafa W , Gamel W , Genedy A and Hassan H (2020) Impact of puerperal sepsis self-care nursing guideline on women’s knowledge and practices. American Journal of Nursing Research 8(2), 132–141. 10.12691/ajnr-8-2-1

[ref23] Nchimbi DB and Joho AA (2022) Puerperal sepsis-related knowledge and reported self-care practices among postpartum women in Dar es Salaam, Tanzania. Women’s health (London, England) 18, 17455057221082954. 10.1177/17455057221082954 PMC9110962 35285367

[ref24] Plante LA , Pacheco LD and Louis JM (2019) SMFM Consult Series: Sepsis during pregnancy and the puerperium. American Journal of Obstetrics and Gynecology 220(4), B2–B10. 10.1016/j.ajog.2019.01.216 30684460

[ref25] Pundir J and Coomarasamy A (2016) Bacterial sepsis following pregnancy. Obstetrics: Evidence-Based Algorithms 64, 292–295. 10.1017/cbo9781107338876.061

[ref26] Seboka K , Gurara AM , Bekele NT , Belachwe YA , Getahun MS and Negussie YM (2024) Determinants of puerperal sepsis among postpartum women at a tertiary care hospital in Ethiopia: An unmatched case-control study. Contraception and Reproductive Medicine 9(1), 18. 38654384 10.1186/s40834-024-00283-x PMC11040802

[ref27] Sultana S , Methe FZ and Faisal Muhammad AAC (2018) Knowledge and practice regarding prevention of puerperal sepsis among postpartum women attending a private hospital. International Journal of Research in Medical Sciences 6(10), 3264–3269.

[ref28] Souza JP , Day LT , Rezende-Gomes AC , Zhang J , Mori R , Baguiya A , Jayaratne K , Osoti A , Vogel JP , Campbell O , Mugerwa KY , Lumbiganon P , Özge T , Cresswell J , Say L , Moran AC and Oladapo OT (2024) A global analysis of the determinants of maternal health and transitions in maternal mortality. The Lancet Global Health 12(2), e306–e316. 10.1016/S2214-109X(23)00468-0 38070536

[ref29] Teferi SM , Terefe B , Temesgen G , Seyoum K , Ejigu Debebe N , Kene C , Geta G and Wedajo LF (2024) Reported self-care practice toward prevention of puerperal sepsis and associated factors among postpartum mothers: Community-based cross-sectional study. SAGE Open Medicine 12, 1–10. 10.1177/20503121241257150 PMC11191623 38911439

[ref30] Tengo GA , Aliyu SA , Endalew ES and Adafirie TN (2025a) Self-care practice and associated factors among postpartum mothers at Karat town, Konso zone, southern Ethiopia. BMC Pregnancy and Childbirth 25(1), 25:700. 10.1186/s12884-025-07821-8 40604615 PMC12224642

[ref31] Tengo GA , Aliyu SA , Endalew ES and Adafirie TN (2025b) Self-care practice and associated factors among postpartum mothers at Karat town, Konso zone, southern Ethiopia. BMC Pregnancy and Childbirth 25(1), 1–10. 10.1186/s12884-025-07821-8 40604615 PMC12224642

[ref32] World Health Organisation (WHO) (2019) WHO Consolidated Guideline on Self-Care Interventions for Health, Geneva: World Health Organization. Available at https://apps.who.int/iris/handle/10665/325480 31334932

[ref33] World Health Organisation, UNICEF, UNFPA (2023) Trends in maternal mortality 2000 to 2020: Estimates by WHO, UNICEF, UNFPA, World Bank Group and UNDESA.

[ref34] World Health Organisation (2017) Statement on Maternal Sepsis: A Leading Cause of Maternal Deaths. Geneva, Switzerland: Department of Reproductive Health and Research, World Health Organisation. WHO/RHR/17.02, pp. 1–4. Available at http://apps.who.int/iris/bitstream/10665/254608/1/WHO-RHR-17.02-eng.pdf

[ref35] Yeneabat T , Hayen A , Getachew T and Dawson A (2022) The effect of national antenatal care guidelines and provider training on obstetric danger sign counselling: A propensity score matching analysis of the 2014 Ethiopia service provision assessment plus survey. Reproductive Health 19(1), 1–14. 10.1186/s12978-022-01442-6 35668529 PMC9167913

